# Digital age triggers: the role of social media evaluation in music performance anxiety

**DOI:** 10.3389/fpsyg.2026.1742379

**Published:** 2026-02-27

**Authors:** Xinying Ma, Litong Lu, Chengdi Luo, Qi Gao

**Affiliations:** 1 The Institute of International Education (IIE), New Era University College, Kuala Lumpur, Malaysia; 2 College of Music, Inje University, Busan, Republic of Korea; 3 College of Agriculture, Guangxi University, Nanning, Guangxi, China; 4 Global Convergence Studies Department, Kangwon National University, Chuncheon, Republic of Korea

**Keywords:** digital performance exposure, emotional regulation, music performance anxiety, online evaluation anxiety, perfectionism, self-compassion, social comparison

## Abstract

This study investigates the mechanisms underlying music performance anxiety (MPA) under conditions of digital visibility, focusing on how digital performance exposure and online evaluation anxiety jointly influence MPA, and how perfectionism and self-compassion moderate these processes. Grounded in social comparison and self-regulation frameworks, structural equation modeling was employed to test the hypothesized pathways among these variables. Results show that digital exposure significantly heightens online evaluation anxiety, which in turn predicts greater MPA, demonstrating a significant mediating pathway. Perfectionism strengthens the association between digital exposure and evaluation anxiety, whereas self-compassion buffers the link between evaluation anxiety and MPA. Multi-group analyses indicate that these structural relations vary by gender and professional identity: female and professional performers report higher sensitivity to evaluative feedback and stronger anxiety responses. These findings suggest that digital visibility broadens the social arena of musical performance while influencing performers’ emotional dynamics. Promoting self-compassion and resilience may support emotional balance and adaptive functioning in increasingly evaluative digital environments.

## 1 Introduction

### 1.1 The global spread and intensification of music performance anxiety in the digital age

Music performance anxiety (MPA) has become a central topic in psychological and educational research over the past two decades. Early studies revealed that MPA affects a wide range of musicians, from adolescent learners to internationally acclaimed performers (Kenny, 2011). Cross-national surveys indicate that approximately 15% to 60% of musicians experience performance anxiety at levels severe enough to interfere with academic achievement, career development, and psychological well-being (Fernholz et al., 2019). This prevalence shows that performance anxiety extends beyond individual differences and represents a global psychological concern shaped by cultural expectations, educational environments, and social evaluation processes. Cross-cultural evidence further demonstrates that the manifestation of performance anxiety is closely related to sociocultural norms. Papageorgi (2021) found that adolescents in Europe and North America tend to report higher anxiety when facing examinations and public performances. In contrast, in many Asian societies, anxiety is more strongly connected to family expectations and collective evaluation. These variations indicate that the sociocultural antecedents of performance anxiety are more complex than earlier assumptions suggested. Moreover, Antonini Philippe et al. (2021) validated the K-MPAI-R scale across multiple languages and identified consistent patterns in three dimensions—cognitive anxiety, physiological tension, and self-doubt. Their results imply that, although cultural factors shape specific expressions of MPA, its fundamental mechanisms remain largely stable across cultural settings, reflecting the global nature of this psychological issue. With the digital transformation of the music industry and the widespread shift toward online dissemination, manifestations of MPA have expanded beyond traditional performance venues into virtual spaces. Gómez López and Sánchez Cabrero (2023) observed that recent intervention studies have begun to examine new triggers of anxiety, including live-streamed performances, social media exposure, and online feedback systems that intensify self-monitoring and cognitive load. Fernholz et al. (2019) also reported that while digital platforms facilitate interaction between musicians and audiences, they simultaneously heighten the risk of continuous evaluation, resulting in more persistent anxiety and shorter recovery periods. In East Asia, Wang and Yang (2024) showed that MPA not only undermines the academic performance of professional music students but also weakens their self-efficacy, thereby reducing career aspirations and sense of fulfillment. Taken together, these findings indicate that MPA has evolved from a temporary emotional reaction into a persistent psychological process influencing music education, career development, and self-identity. The challenge now lies in understanding how traditional psychological mechanisms interact with the digital social environment. Addressing this issue requires theoretical and empirical frameworks capable of explaining the dynamic and cross-cultural nature of performance anxiety in the digital era.

### 1.2 From traditional performance anxiety to digital evaluation stress: the evolution of theoretical paradigms

The theoretical understanding of Music Performance Anxiety (MPA) has gradually shifted from an individual and clinical focus to frameworks that situate the phenomenon within broader social and digital contexts. During the 1990s, scholars began to reassess its conceptual foundations. Through a critical review of earlier work, Brodsky (1996) argued that initial studies had overemphasized clinical anxiety and physiological reactions while overlooking the psychological structure of performance as a social act and an aspect of identity formation. He suggested that MPA should be viewed as a psychological condition shaped by interactions among social evaluation, professional identity, and self-concept. This approach moved the field beyond the limits of clinical diagnosis and provided a new theoretical orientation for examining performance anxiety from social and cognitive perspectives. In the early twenty-first century, research attention moved from describing pathological symptoms toward exploring the psychological mechanisms underlying performance anxiety. Burin and Osório (2017), in their review of the etiology of MPA, proposed that its causes should be understood through the integration of personality traits, cognitive biases, and social context. They emphasized that anxiety originates not only in internal emotional responses but also in cultural expectations, audience evaluations, and performers’ sense of self-efficacy. This period represented a conceptual transition from single-factor explanations to multidimensional interactive models that underscored the role of sociocultural context in shaping anxiety. Extending this line of inquiry, Guyon et al. (2020) introduced the Challenge and Threat Framework, explaining from a psychophysiological standpoint how performers appraise stress in performance contexts. When pressure is perceived as an opportunity to demonstrate ability, it tends to produce positive mobilization and moderate arousal; when it is viewed as a threat, it triggers anxiety and avoidance. The model advanced MPA research by shifting attention from static description to dynamic process analysis and by integrating situational appraisal, cognitive processing, and physiological activation within a coherent theoretical framework. Twitchell et al. (2025), working within the stress theoretical framework, conducted a comprehensive review of MPA research and argued that traditional models cannot fully explain the persistence and recurrence of anxiety in the digital era. They conceptualized MPA as a multifaceted psychological process encompassing cognitive, emotional, and social dimensions, shaped by the combined influences of evaluative pressure, attentional control, and self-regulation. This reconceptualization represented a move from individual-level explanations to an integrative multi-system model. In parallel, Kinney et al. (2025), reviewing intervention studies, argued that future theoretical frameworks must account for contextual diversity—particularly the psychological burdens created by continuous evaluation on digital platforms and online performances. They observed that as digital media became deeply integrated into music education and professional practice, MPA has expanded beyond the traditional stage, showing chronic and persistent patterns that require renewed theoretical interpretation. The recent work of Musgrave et al. (2025) further developed this trajectory by introducing the concept of digital evaluation stress. They argued that social media platforms, through likes, comments, and algorithmic recommendations, place musicians in a state of continuous social visibility. Sustained online exposure reinforces self-monitoring and social comparison, which can trigger or intensify performance anxiety. This study was one of the earliest attempts to link MPA with the psychological dynamics of digital society, illustrating how anxiety adapts to new evaluative environments and broadening the conceptual reach of music psychology. Overall, the evolution of MPA theory demonstrates a movement from individual-based emotion regulation models toward analyses of psychological adaptation within social and technological settings. As digital platforms reshape the relationship between performance and evaluation, MPA is increasingly recognized as a multifaceted psychological phenomenon. Current research trends converge on an integrative model that connects digital evaluation, emotional response, and psychological regulation, aiming to clarify how musicians adapt psychologically in digital performance contexts.

### 1.3 The psychological mechanisms of social media evaluation pressure: a chain pathway from digital exposure to music performance anxiety

Research on music performance anxiety (MPA) has gradually moved from an emotion-centered perspective to models that link anxiety with social and technological contexts. Early studies emphasized that anxiety should not be seen only as a negative reaction but as an emotion shaped by regulation. Murphy et al. (2024) reported that when musicians feel anxiety together with positive emotions, it can serve a motivating role that improves focus and performance quality. When anxiety involves self-threatening thoughts, however, its effect becomes inhibitory rather than facilitative. This “facilitation–impairment” dual-pathway model shows that emotional context plays a key role in regulating anxiety, yet most research still focuses on offline performance settings and rarely considers how digital evaluation changes this process. Guyon et al. (2020) used the challenge–threat framework to show that performers’ appraisals of social evaluation lead to different psychological outcomes. When evaluation is viewed as a chance to display ability, it promotes confidence and positive arousal; when it is viewed as a threat, it produces anxiety and avoidance. This framework helps explain how evaluative pressure works in performance situations, including those shaped by social media. Irie et al. (2023) examined how MPA change over time. They found that anxiety increases before a performance, peaks during it, and may rise again afterward as performers reflect on their mistakes. Without effective regulation, anxiety can be strengthened through repeated performances and self-comparison. In environments with constant social media exposure, such patterns may become stable rather than short-lived. Hejjawi (2023) studied piano learners and found that social media reactions such as likes, comments, and follower responses influence self-worth and anxiety. Positive feedback can raise self-esteem for a short time but may also create reliance on external approval, reducing intrinsic motivation and increasing conflict in self-monitoring. Musgrave et al. (2025) observed a similar pattern: long-term engagement with social media encourages an algorithm- and audience-oriented mindset in which musicians feel pressure to produce content continuously. This constant exposure reinforces cycles of comparison and self-evaluation, shifting anxiety from the stage to online spaces and making it more persistent. Previous research has clarified how performance anxiety depends on emotional regulation, physiological control, and time-related processes, but several gaps remain. First, most studies examine traditional settings and overlook the psychological pressure created by continuous social media evaluation. Second, emotional regulation, social evaluation, and digital exposure have rarely been examined within a single framework. Third, individual factors such as perfectionism and self-compassion are not well studied in digital contexts. To address these gaps, this study develops a sequential model linking “social media exposure → online evaluation anxiety → music performance anxiety.” Perfectionism and self-compassion are included as moderators to explain how performance anxiety develops and is managed in digital environments. The model is expected to contribute to both theory and practice in music psychology by clarifying psychological adjustment processes under digital evaluation.

In previous research, numerous studies have supported the view that social media use constitutes an important mental health risk context for adolescents and young adults. In particular, McComb et al. (2023) demonstrated, through a meta-analytic review, that exposure to social media comparison systematically undermines self-evaluations and emotional well-being. Accordingly, the present study focuses on a salient psychological process in digital performance contexts, namely evaluation-based digital visibility, and examines how digital performance exposure increases sensitivity to external feedback, leading to online evaluation anxiety, which in turn intensifies music performance anxiety. In addition, two individual difference variables, perfectionism and self-compassion, are introduced to examine how different self-regulatory tendencies shape the transformation of digital evaluation pressure into performance-related anxiety. Perfectionism is typically associated with heightened self-demand and strong concern for others’ evaluations, which may amplify anxiety responses in evaluative contexts, whereas self-compassion reflects an individual’s capacity for acceptance and awareness when facing pressure or mistakes and may help buffer the negative emotional impact of evaluation. Methodologically, this study employs structural equation modeling to examine the relationships among digital performance exposure, online evaluation anxiety, and music performance anxiety, and to test a comprehensive model incorporating both mediating and moderating pathways. Furthermore, given potential differences in sensitivity to social evaluation and performance related identity risks, this study explores whether the proposed structural relationships differ across gender and performer identity, specifically between students and professional musicians.

## 2 Literature review

### 2.1 Theoretical foundations and psychological mechanisms of music performance anxiety

Research on music performance anxiety (MPA) has developed into a field that brings together emotional, physiological, and cognitive perspectives. Recent studies examine how these processes interact and differ across individuals and performance situations. Early research described MPA mainly as an emotional reaction to the pressure of performing on stage. More recent work shows that anxiety is closely tied to how musicians perceive themselves and how they believe others evaluate them. Castiglione et al. (2018) compared professional and amateur musicians and found that self-schema and perceived self-efficacy directly influence anxiety during performance. When the gap between a musician’s ideal and actual self-increases, anxiety becomes more intense. These results indicate that MPA reflects inner conflict related to self-identity rather than a simple response to situational stress. Racine et al. (2025) developed the Excellence–Perfectionism Model, which distinguishes between adaptive striving for excellence and self-critical perfectionism. They found that self-critical tendencies are strongly related to higher anxiety, while growth-oriented striving supports greater performance stability. These findings show that motivational orientation affects how MPA develops and clarifies how perfectionistic traits relate to anxiety in performance settings. Osborne and Kirsner (2022) reviewed studies on the main mechanisms of MPA and concluded that it results from the combined influence of perceived social threat, physiological arousal, and cognitive bias. They argued that emotional responses should be understood within their social and cultural settings. Guyon et al. (2020) tested the challenge–threat model with physiological data and found consistent links between heart rate variability, cortisol levels, and reported anxiety. When performers interpret pressure as a challenge, their physiological and psychological states remain adaptive; when they view it as a threat, tension rises, and performance tends to decline. Spahn et al. (2023) distinguished between trait and state MPA. Trait MPA is associated with enduring personality characteristics and long-term psychological patterns, whereas state MPA depends on factors such as performance frequency, audience type, and task difficulty. Zhukov (2019) argued that MPA stems not only from perceived pressure but also from how performers interpret and handle it. Managing anxiety therefore, requires combining psychological training with supportive environments to strengthen self-regulation. Taken together, these studies show a continuing shift from single-factor explanations to broader models that integrate self-cognition, motivation, social evaluation, and physiological arousal. Many scholars now regard MPA as a cognitive–emotional process grounded in self-evaluation and shaped by social and cultural experience. This integrated view offers a solid basis for understanding how performance anxiety arises and how it can be addressed in digital performance environments.

### 2.2 Social evaluation and comparison in the digital age: the reconstructed performance environment

The rise of digital media has changed the conditions in which performers compare themselves with others and seek evaluation. The interactive design and algorithmic systems of social platforms are now reshaping how musicians experience and interpret their performances. A meta-analysis by McComb et al. (2023) showed that exposure to upward comparison targets on social media lowers self-evaluations and emotional well-being, an effect especially strong among performance-oriented groups. Tian et al. (2025) found that upward social comparison on social media increases appearance-related anxiety and weakens overall mental health through indirect psychological processes. Individuals with higher self-efficacy, however, are better able to buffer these negative effects. Andrade et al. (2023) used a daily diary method and observed that people who often compare themselves to others report lower everyday well-being, but brief self-affirmation exercises can lessen this decline. Okano and Nomura (2023) examined this issue in relation to social anxiety and found that self-perceived social competence moderates the link between comparison orientation and anxiety. People with stronger social anxiety tend to evaluate themselves more negatively in online environments. Chen and Toma (2025) reported that self-affirmation helps to reduce the emotional impact of upward comparison. It allows individuals to maintain a stable sense of self and better emotional balance under online evaluation. In research on musicians, Musgrave et al. (2025) found that those who stay under constant online visibility often tie their sense of self-worth to audience feedback. Over time, their creative drive shifts from personal expression to dependence on algorithmic reactions—a pattern they described as a “content-factory” mindset. The speed of online feedback, the visibility of engagement metrics, and the lasting nature of digital exposure have changed how performance is experienced. These factors keep musicians under continuous evaluation and heighten anxiety and self-monitoring. Overall, the evidence shows that social comparison and evaluation in digital environments have expanded the scope of music performance anxiety and changed how performers understand performance itself. Through algorithmic control and public feedback, digital visibility is redefining musicians’ sense of success, identity, and authenticity in their creative work.

### 2.3 Mediation and moderation mechanisms: from online evaluation anxiety to individual differences

Recent research on music performance anxiety (MPA) increasingly focuses on how mediation and moderation processes explain individual psychological differences. Anxiety is no longer viewed as a single emotional response but as a dynamic process shaped by factors such as perceived risk, self-compassion, perfectionism, and resilience. Yang et al. (2025) found that musicians’ professional competence affects performance anxiety indirectly through perceived psychological risk, with resilience moderating this link. High resilience helps reduce anxiety arising from ability-related pressure, showing how competence, perceived risk, and anxiety are connected. Perfectionism is a relatively stable cognitive trait that often acts as a mediator in the development of performance anxiety. Xiong et al. (2024) reported that maladaptive perfectionism heightens anxiety through poor emotional regulation, while self-compassion weakens this effect. Akkus et al. (2023) also found that self-compassion mediates the link between perfectionism and social anxiety, suggesting that a kinder attitude toward oneself can interrupt habitual self-criticism. Building on this line of work, Benedetto et al. (2024) showed that self-compassion not only lowers anxiety among perfectionists but also promotes psychological balance by improving subjective well-being. Tan (2023) examined how self-compassion and resilience interact across cultural contexts and found that among people with high perfectionistic tendencies, self-acceptance improves coping ability and lowers the risk of anxiety. Reviewing studies on social media use, Manjanatha et al. (2025) observed that self-compassion generally protects against the negative emotional effects of digital stress and external evaluation. Taken together, these findings indicate that performance anxiety develops through multiple interacting pathways. Perceived risk mediates the link between ability and anxiety, whereas resilience and self-compassion buffer stress and perfectionistic pressure. In digital environments, where evaluation and comparison are constant, these pathways may become more pronounced, producing greater variation across individuals. Future studies should further examine how personal traits shape these mediating and moderating effects and how interventions that enhance self-compassion and resilience can help reduce performance-related anxiety.

### 2.4 Toward an integrative model of anxiety in the digital age

The study of music performance anxiety (MPA) in the digital era has begun to integrate psychological resources, social cognition, and media environments within a unified framework. Earlier models mainly addressed stage pressure and physiological arousal, but in the age of social media and online exposure, anxiety has become more continuous, context-specific, and socially driven. Twitchell et al. (2025) pointed out that classical stress models overlook how stress accumulates and is re-experienced in digital settings. They proposed adding dynamic appraisal and social exposure factors to make these models more adaptive. Lim and Dhamabutra (2025) likewise argued that performance anxiety should be seen as a psychological system linking cognitive appraisal, emotional regulation, and social interaction. They noted that older clinical views fail to reflect the ongoing evaluative pressures typical of digital communication. Drawing on positive psychology, Jiang and Tong (2024) suggested that psychological capital—such as self-esteem and flow—mediates the link between digital exposure and anxiety, helping to explain individual differences in resilience. Barros et al. (2024) provided cross-cultural evidence showing that frequent external evaluation, intensive social media engagement, and performance self-efficacy together foster a socially spreading form of anxiety, especially among young music students. Musgrave et al. (2025) identified algorithmic recommendation systems and the fan economy as elements of a “content pressure environment,” where constant visibility and repeated self-comparison sustain recurring anxiety. Gómez López and Sánchez Cabrero (2023) reviewed recent intervention approaches and recommended combining cognitive-behavioral methods, mindfulness, self-compassion, and digital-literacy training to meet the complex psychological demands of digital performance. Building on this work, the present study develops a digital-era anxiety model organized around three main dimensions: psychological capital, social evaluation, and digital exposure. The model outlines a sequence connecting digital stress, cognitive threat appraisal, psychological resources, and anxiety regulation. It explains how social media evaluation influences MPA through cognitive and self-regulatory processes and examines how perfectionism and self-compassion moderate these effects. This framework provides a practical basis for improving musicians’ mental health and for designing educational strategies suited to digitally mediated performance settings.

Based on social comparison and self-regulation perspectives, the following hypotheses are proposed:

*H1*: Digital performance exposure is positively associated with online evaluation anxiety.

*H2*: Online evaluation anxiety is positively associated with music performance anxiety.

*H3*: Online evaluation anxiety mediates the relationship between digital performance exposure and music performance anxiety.

*H4*: After accounting for online evaluation anxiety, digital performance exposure remains a significant predictor of music performance anxiety, indicating partial mediation.

To further capture individual differences in responses to digital evaluative contexts, the following moderation hypotheses are proposed:

*H5*: Perfectionism moderates the relationship between digital performance exposure and online evaluation anxiety, such that the association is stronger among individuals with higher levels of perfectionism.

*H6*: Self compassion moderates the relationship between online evaluation anxiety and music performance anxiety, such that the association is weaker among individuals with higher levels of self-compassion.

## 3 Methods

### 3.1 Study design

This study employed a cross-sectional survey design to examine the associations among digital performance exposure, online evaluation anxiety, and music performance anxiety, as well as the moderating roles of perfectionism and self-compassion. Data were collected through an anonymous online questionnaire administered via Wenjuanxing, a widely used online survey platform in China,^1^ and distributed to individuals who reported prior experience with digital or online musical performance contexts. The study focused on identifying direct, indirect, and conditional relationships among the variables using structural equation modeling.

### 3.2 Participants

Participants included university students and professional musicians with experience in digital or online performance. A combination of purposive sampling and snowball recruitment was used to reach individuals engaged in digital performance activities. The survey was distributed through online platforms associated with universities, music schools, and virtual performance communities. A total of 382 questionnaires were returned, and after excluding incomplete or invalid responses, 320 valid questionnaires were retained, yielding an effective response rate of 83.8%. Participants ranged in age from 18 to 25 years (M = 20.98, SD = 1.44), with a balanced gender distribution (49.38% male, 50.62% female). With respect to performer identity, 72.19% of participants were university students and 27.81% were professional musicians.

### 3.3 Procedure

Data were collected through an anonymous online survey. Before completing the questionnaire, all participants were presented with an electronic informed consent form explaining the purpose of the study, the voluntary nature of participation, and the confidentiality of responses. Only participants who provided consent were able to proceed to the survey. The questionnaire included measures of digital performance exposure, online evaluation anxiety, music performance anxiety, perfectionism, and self-compassion, as well as basic demographic information. All responses were self-reported, and no personally identifiable information was collected.

### 3.4 Measures

All study variables were assessed using self-report questionnaires. Unless otherwise specified, items were rated on five-point Likert scales ranging from 1 (strongly disagree) to 5 (strongly agree), while music performance anxiety was measured using a seven-point Likert scale to assess anxiety intensity across a broader response range. Digital performance exposure was measured using two indicators assessing the frequency of online performance activities and reliance on audience feedback during digital performances. Online evaluation anxiety was assessed using two indicators reflecting fear of negative evaluation and sensitivity to online comments and feedback. Music performance anxiety was measured using two indicators capturing cognitive worry and emotional–physiological tension related to performance. Perfectionism was assessed using indicators reflecting concern over mistakes and perceived external expectations, whereas self-compassion was measured using indicators reflecting self-kindness and mindful awareness in response to difficulty or failure. All indicators were treated as observed variables in the confirmatory factor analysis and structural equation modeling, and the reliability and construct validity of all measures were evaluated prior to hypothesis testing. All items were adapted from prior validated instruments in the performance anxiety and self-regulation literature, with wording adjusted to fit digital performance contexts.

### 3.5 Data analysis

Data analyses were conducted using SPSS 26.0 and AMOS 26.0. Confirmatory factor analysis was first performed to examine the measurement model and assess construct validity. Internal consistency was evaluated using Cronbach’s alpha, composite reliability, and average variance extracted. Structural equation modeling was then used to test the direct and indirect relationships among variables. Mediation effects were examined using bootstrap procedures with 5,000 resamples to estimate confidence intervals. Moderation effects were tested by including interaction terms in the structural model. Multi-group analyses were conducted to explore potential differences across gender and performer identity (students versus professional musicians).

## 4 Results

### 4.1 Reliability and confirmatory factor analysis (CFA)

Before testing the hypothesized structural relationships, we conducted a confirmatory factor analysis (CFA) to evaluate the measurement model and to ensure that all constructs demonstrated adequate reliability and convergent validity.

Table 1 shows the reliability indices and confirmatory factor analysis (CFA) results for five latent variables: digital performance exposure, online evaluation anxiety, music performance anxiety, perfectionism, and self-compassion. The standardized factor loadings for all items ranged from 0.75 to 0.88 and were statistically significant (*p* < 0.001). Cronbach’s *α* values ranged from 0.88 to 0.93, and all composite reliability (CR) coefficients were above 0.87. The average variance extracted (AVE) ranged from 0.62 to 0.68, indicating strong internal consistency and satisfactory convergent validity across all measures. The measurement model was stable, and the results help clarify how performance anxiety is organized psychologically in digital social contexts. High factor loadings for digital performance exposure and online evaluation anxiety point to a close link between individuals’ online performance behavior and their social interactions. Continued exposure to evaluation appears to strengthen feelings of being observed and compared, turning temporary stage tension into enduring social-cognitive pressure. Among the five constructs, music performance anxiety showed the highest reliability (*α* = 0.93), reflecting the strong interaction between cognitive worry and physiological arousal. Perfectionism and self-compassion displayed opposite patterns. Perfectionism increased internal stress through fear of mistakes and concern with external expectations, whereas self-compassion reduced evaluative anxiety through acceptance and mindfulness. This difference highlights distinct psychological pathways involved in the regulation of performance anxiety.

**Table 1:** Reliability and CFA results (*N* = 320).

Latent variable	Indicator	Standardized loading	Cronbach’s α	AVE	CR
Digital performance exposure (A)	A1 Online performance frequency	0.81***	0.88	0.63	0.87
	A2 Evaluation dependence	0.84***			
Online evaluation anxiety (B)	B1 Fear of being evaluated	0.85***	0.91	0.68	0.9
	B2 Comment sensitivity	0.88***			
Music performance anxiety (C)	C1 Cognitive worry	0.87***	0.93	0.65	0.91
	C2 Emotional–physiological tension	0.86***			
Perfectionism (D)	D1 Error anxiety	0.78***	0.89	0.62	0.88
	D2 Perceived others’ expectations	0.80***			
Self compassion (E)	E1 Self kindness	0.75***	0.9	0.66	0.89
	E2 Mindful awareness	0.83***			

Note: Statistical significance is indicated as follows: * *p* < 0.05, ** *p* < 0.01, and *** *p* < 0.001.

### 4.2 Descriptive statistics

Table 2 presents the descriptive statistics and bivariate correlations among the study variables to provide an overview of their distributions and preliminary associations prior to the structural analyses.

**Table 2:** Descriptive statistics and correlations (*N* = 320).

Variable	M	SD	A	B	C	D	E
A Digital performance exposure	3.68	0.72					
B Online evaluation anxiety	3.92	0.67	0.48***				
C Music performance anxiety	3.79	0.7	0.46***	0.54***			
D Perfectionism	3.54	0.74	0.33***	0.37***	0.42***		
E Self compassion	3.21	0.76	−0.22**	−0.28***	−0.31***	−0.19**	

Note: Statistical significance is indicated as follows: * *p* < 0.05, ** *p* < 0.01, and *** *p* < 0.001.

Table 2 presents the descriptive statistics and correlations among the main variables. Online evaluation anxiety (M = 3.92, SD = 0.67) and music performance anxiety (M = 3.79, SD = 0.70) showed relatively higher mean levels in the sample. Digital performance exposure (M = 3.68, SD = 0.72) was positively correlated with online evaluation anxiety (*r* = 0.48, *p* < 0.001) and music performance anxiety (*r* = 0.46, *p* < 0.001). The strongest association was observed between online evaluation anxiety and music performance anxiety (*r* = 0.54, *p* < 0.001). Perfectionism was positively correlated with online evaluation anxiety and music performance anxiety (*r* = 0.33–0.42, *p* < 0.001), whereas self-compassion was negatively correlated with both anxiety measures (*r* = −0.22 to −0.31, *p* < 0.01). Overall, the correlation patterns were consistent with the proposed relationships and provided preliminary support for subsequent structural analyses.

### 4.3 Structural equation model and Main path analysis

**Figure 1:**
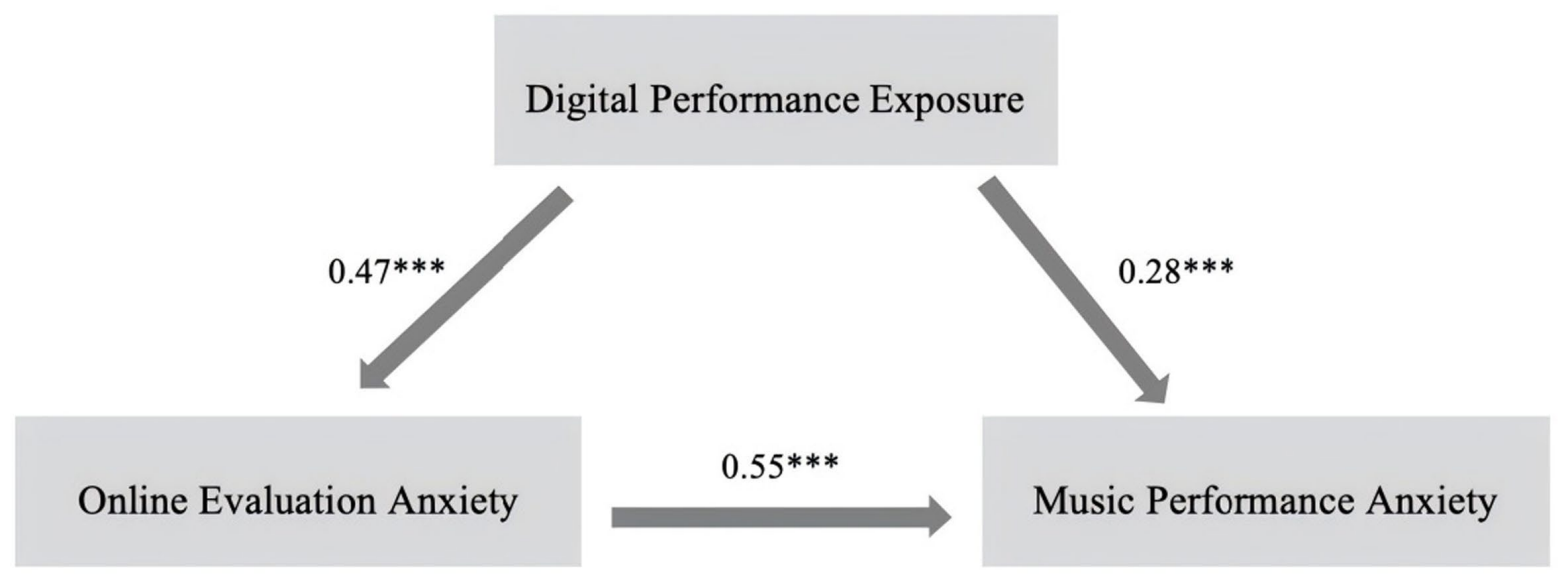
Structural equation model (main paths). Note: Statistical significance is indicated as follows: * *p* < 0.05, ** *p* < 0.01, and *** *p* < 0.001.

Table 3 and Figure 1 present the main path results of the structural equation model, describing the relationships among digital performance exposure, online evaluation anxiety, and music performance anxiety (MPA). These paths were estimated to test the hypothesized direct and indirect associations among the three core variables. The results showed that digital performance exposure significantly predicted online evaluation anxiety (*β* = 0.47, *t* = 8.02, *p* < 0.001), and that online evaluation anxiety, in turn, significantly predicted MPA (*β* = 0.55, *t* = 9.18, *p* < 0.001). In addition, digital performance exposure exhibited a significant direct effect on MPA (*β* = 0.28, *t* = 3.91, *p* < 0.001). Together, these results provide empirical support for Hypotheses 1 and 2 and indicate that the association between digital performance exposure and music performance anxiety is not fully accounted for by online evaluation anxiety. The simultaneous significance of both the indirect pathway through online evaluation anxiety and the direct pathway from digital performance exposure to MPA indicates that online evaluation anxiety explains a substantial portion, but not the entirety, of this association. This pattern is consistent with a partial mediation structure, which is further examined in the subsequent mediation analysis. At the level of the structural results, the findings indicate that higher levels of digital performance exposure are associated with increased online evaluation anxiety and elevated music performance anxiety, while online evaluation anxiety functions as a central intermediate variable linking exposure to anxiety outcomes. The persistence of a significant direct effect suggests that additional variance in music performance anxiety remains associated with digital performance exposure beyond the contribution of online evaluation anxiety.

**Table 3:** SEM path analysis (*N* = 320).

Path	*β*	SE	*t*	*p*	Sig.
DPE → OEA	0.47	0.06	8.02	<0.001	***
OEA → MPA	0.55	0.05	9.18	<0.001	***
DPE → MPA	0.28	0.07	3.91	<0.001	***

Note: Statistical significance is indicated as follows: * *p* < 0.05, ** *p* < 0.01, and *** *p* < 0.001.

### 4.4 Mediating effect of online evaluation anxiety (bootstrap)

Table 4 reports the mediating role of online evaluation anxiety in the relationship between digital performance exposure and music performance anxiety (MPA). This analysis was conducted to formally test the hypothesized mediation pathway proposed in Hypothesis 3. The bootstrap results showed that the direct effect of digital performance exposure on MPA was significant [*β* = 0.28, 95% CI (0.14, 0.40), *p* < 0.001], and that the indirect effect through online evaluation anxiety was also significant [*β* = 0.26, 95% CI (0.17, 0.36), *p* < 0.001]. The total effect of digital performance exposure on MPA reached significance as well [*β* = 0.54, 95% CI (0.42, 0.65), *p* < 0.001]. The confidence interval for the indirect effect did not include zero, providing support for Hypothesis 3, while the continued significance of the direct effect is consistent with Hypothesis 4. Taken together, these results indicate that the association between digital performance exposure and music performance anxiety operates through both direct and indirect pathways, with online evaluation anxiety accounting for a substantial proportion of the total effect. At the result level, the findings show that increased digital performance exposure is associated with higher music performance anxiety, partly through elevated online evaluation anxiety, while a significant direct association remains after accounting for the mediator. This pattern supports a partial mediation structure and indicates that online evaluation anxiety functions as a central intermediate variable linking digital performance exposure to music performance anxiety.

**Table 4:** Bootstrap mediation results.

Effect	*β*	SE	95% CI	*p*	Sig.
Direct (DPE → MPA)	0.28	0.07	(0.14, 0.40)	<0.001	***
Indirect (DPE → OEA → MPA)	0.26	0.05	(0.17, 0.36)	<0.001	***
Total effect	0.54	0.06	(0.42, 0.65)	<0.001	***

Note: Statistical significance is indicated as follows: * *p* < 0.05, ** *p* < 0.01, and *** *p* < 0.001.

### 4.5 Moderating effect of perfectionism

**Figure 2:**
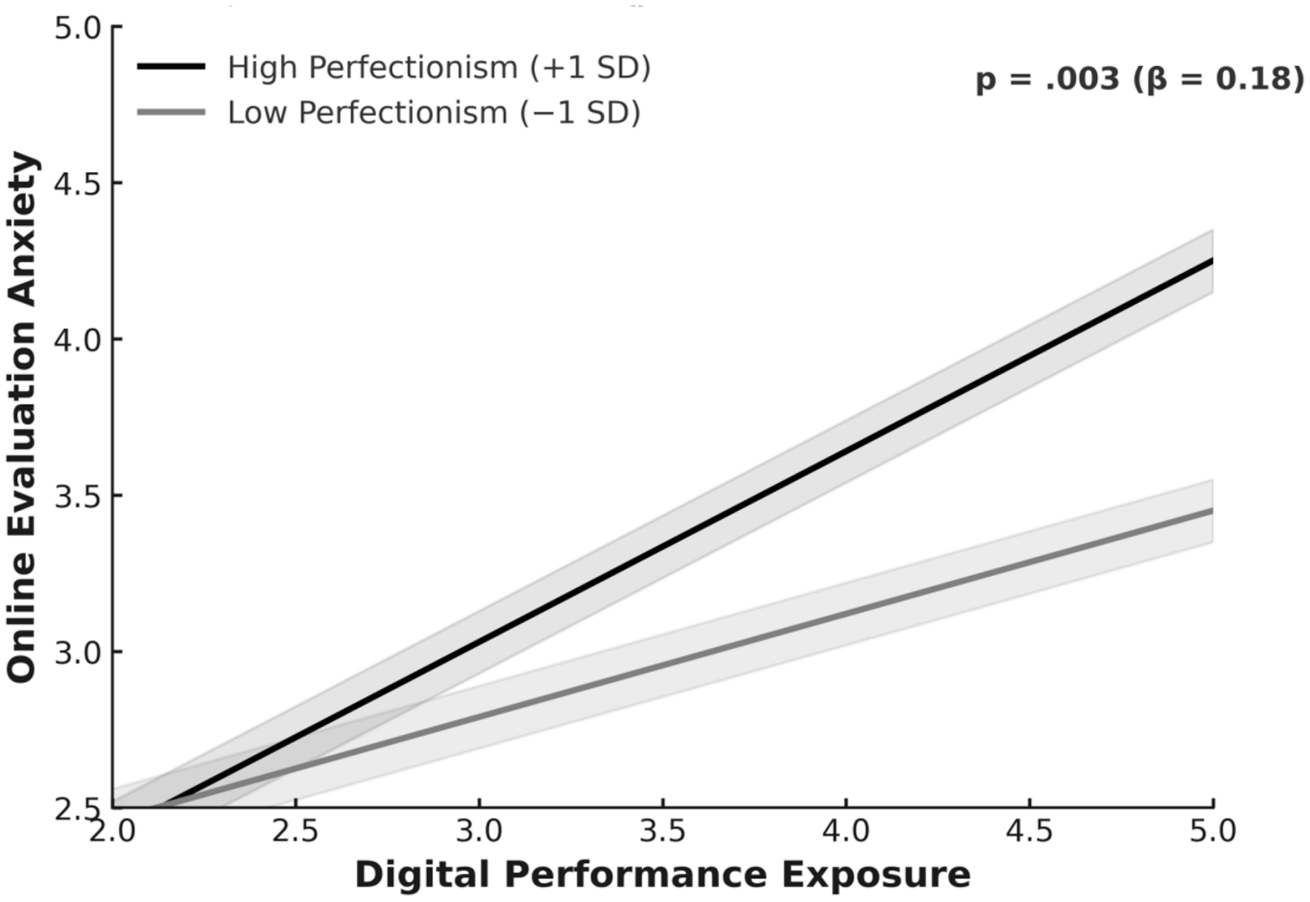
Moderating effect of perfectionism.

Table 5 and Figure 2 present the results of the moderation analysis examining the role of perfectionism in the relationship between digital performance exposure (DPE) and online evaluation anxiety (OEA). This analysis was conducted to test the moderation effect specified in Hypothesis 5. The interaction term between digital performance exposure and perfectionism was statistically significant (*β* = 0.18, *t* = 3.02, *p* = 0.003), indicating that the association between digital performance exposure and online evaluation anxiety varies as a function of perfectionism. This significant interaction provides empirical support for Hypothesis 5, demonstrating that higher levels of perfectionism strengthen the positive association between digital performance exposure and online evaluation anxiety. As illustrated in Figure 2, individuals with higher perfectionism showed a steeper increase in online evaluation anxiety as digital performance exposure increased, whereas those with lower levels of perfectionism exhibited a more gradual increase. At the result level, this pattern indicates that perfectionism moderates the strength of the relationship between digital performance exposure and online evaluation anxiety, such that the impact of exposure on evaluative anxiety is more pronounced among individuals with higher perfectionistic tendencies. The divergence between the two trend lines in Figure 2 reflects systematic differences in the magnitude of the exposure–anxiety association across levels of perfectionism.

**Table 5:** Moderating effect of perfectionism.

Path	*β*	SE	*t*	*p*	Sig.
DPE × PER → OEA	0.18	0.06	3.02	0.003	**
DPE → OEA	0.47	0.06	8.1	<0.001	***
PER → OEA	0.33	0.08	4.12	<0.001	***

Note: Statistical significance is indicated as follows: * *p* < 0.05, ** *p* < 0.01, and *** *p* < 0.001.

### 4.6 Moderating effect of self compassion

**Figure 3:**
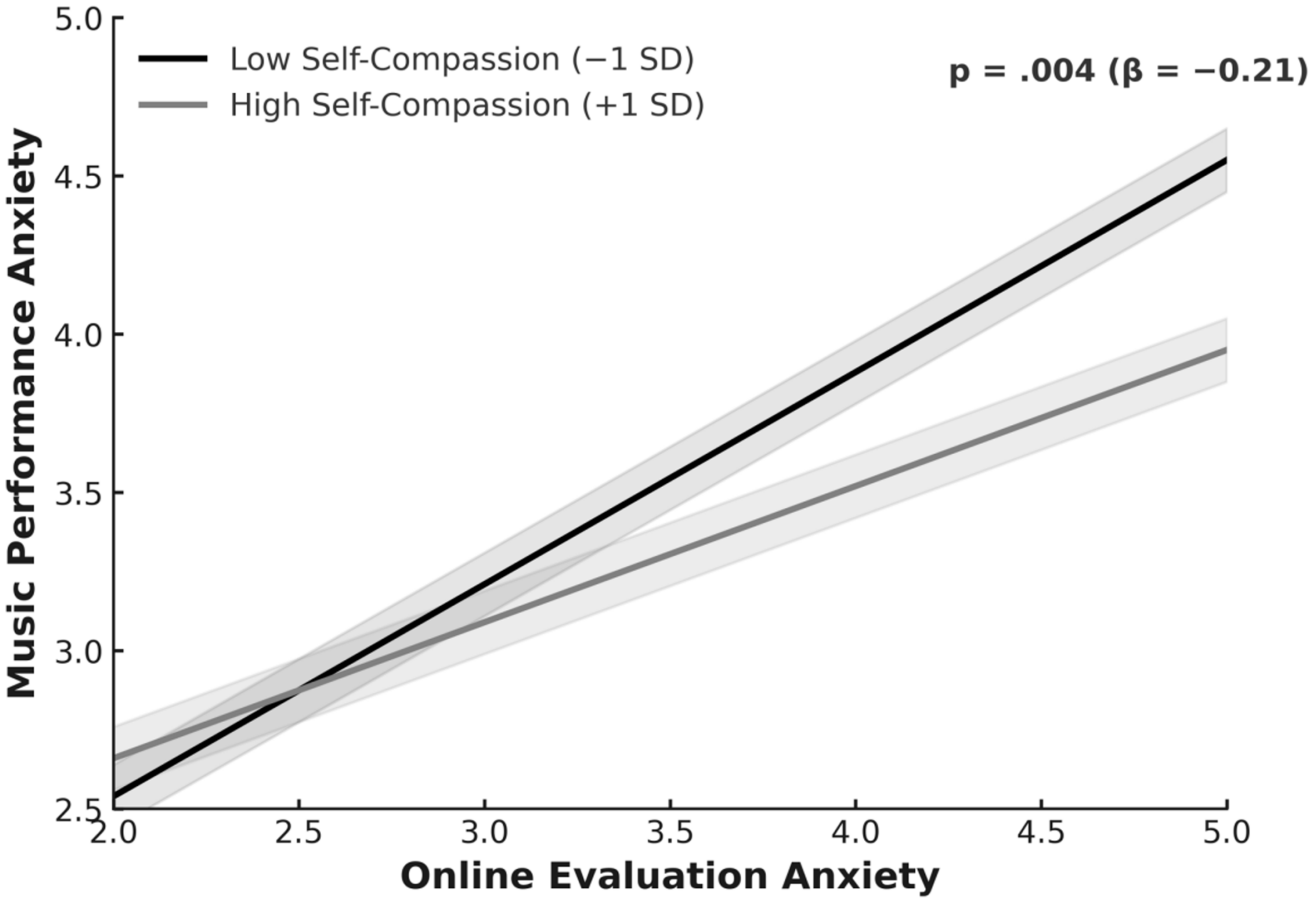
Moderating effect of self compassion.

Table 6 and Figure 3 present the results of the moderation analysis examining the role of self-compassion in the relationship between online evaluation anxiety (OEA) and music performance anxiety (MPA). This analysis was conducted to test the buffering moderation effect proposed in Hypothesis 6. The results showed that online evaluation anxiety significantly predicted music performance anxiety (*β* = 0.55, *p* < 0.001), while self-compassion was negatively associated with music performance anxiety (*β* = −0.24, *p* = 0.003). Importantly, the interaction between online evaluation anxiety and self-compassion was statistically significant (*β* = −0.21, *p* = 0.004), indicating that the strength of the association between online evaluation anxiety and music performance anxiety varied across levels of self-compassion. This significant interaction provides empirical support for Hypothesis 6, demonstrating that higher levels of self-compassion attenuate the positive association between online evaluation anxiety and music performance anxiety. As illustrated in Figure 3, individuals with lower self-compassion exhibited a steeper increase in music performance anxiety as online evaluation anxiety increased, whereas those with higher self-compassion showed a more gradual slope. At the result level, this pattern indicates that self-compassion moderates the magnitude of the association between online evaluation anxiety and music performance anxiety, such that the impact of evaluative anxiety on performance-related anxiety is weaker among individuals with higher self-compassion.

**Table 6:** Moderating effect of self compassion.

Path	*β*	SE	*t*	*p*	Sig.
OEA × SC → MPA	−0.21	0.07	−2.93	0.004	**
OEA → MPA	0.55	0.05	9.23	<0.001	***
SC → MPA	−0.24	0.08	−3.00	0.003	**

Note: Statistical significance is indicated as follows: * *p* < 0.05, ** *p* < 0.01, and *** *p* < 0.001.

### 4.7 Robustness check and multi group comparison

Table 7 presents the results of the multi-group path analysis conducted to examine the robustness of the proposed structural model across gender and performer identity. The analysis showed that the structural paths among digital performance exposure (DPE), online evaluation anxiety (OEA), and music performance anxiety (MPA) remained statistically significant across all subgroups, although the magnitude of these relationships varied. Specifically, among female participants, the path coefficients from DPE to OEA (*β* = 0.52, *p* < 0.001) and from OEA to MPA (*β* = 0.59, *p* < 0.001) were stronger than those observed among male participants (*β* = 0.43 and *β* = 0.46, respectively), while the direct effect of DPE on MPA among males remained significant but weaker (*β* = 0.22, *p* < 0.05). Differences were also observed across performer identity groups. For university students, the association between DPE and OEA was moderate (*β* = 0.41, *p* < 0.01), whereas for professional musicians this association was stronger (*β* = 0.52, *p* < 0.001). Despite these variations in path strength, the overall model demonstrated satisfactory fit across all groups (CFI = 0.952–0.955, TLI = 0.943–0.948, RMSEA = 0.044–0.047). Taken together, these results indicate that the proposed structural relationships are robust across gender and performer identity, while the magnitude of key pathways differs systematically across subgroups.

**Table 7:** Multi group path comparison.

Group	DPE → OEA	OEA → MPA	DPE → MPA	Model fit (CFI/TLI/RMSEA)
Male	0.43**	0.46**	0.22*	0.952/0.943/0.047
Female	0.52***	0.59***	0.28**	0.953/0.945/0.046
Student	0.41**	0.51***	0.25**	0.954/0.947/0.045
Professional musician	0.52***	0.55***	0.29**	0.955/0.948/0.044

Note: Statistical significance is indicated as follows: * *p* < 0.05, ** *p* < 0.01, and *** *p* < 0.001.

## 5 Discussion

### 5.1 Digital performance exposure, online evaluation anxiety, and music performance anxiety

The results indicate that digital performance environments reshape music performance anxiety through a process that is neither purely detrimental nor uniformly adaptive. Greater digital performance exposure was associated with higher levels of online evaluation anxiety, supporting Hypothesis 1 and suggesting that sustained visibility heightens performers’ sensitivity to being judged. This association likely reflects an increase in anticipatory concern and self-monitoring, as performers become aware that their performances may be evaluated continuously rather than at discrete moments. Such heightened awareness can make evaluative pressure more salient and emotionally taxing, particularly for individuals who are strongly invested in external feedback or audience perception. At the same time, increased evaluative awareness does not necessarily signify maladjustment alone. In digitally mediated performance contexts, constant visibility and audience feedback have become normative rather than exceptional conditions. For some performers, especially those accustomed to online platforms or professional performance cultures, heightened sensitivity to evaluation may represent an adaptive adjustment to prevailing performance norms, in which vigilance and preparedness are required to navigate ongoing exposure. Consistent with Hypothesis 2, online evaluation anxiety was positively related to music performance anxiety, indicating that concern about being evaluated functions as a key psychological pathway through which digital visibility translates into performance-related distress. This finding suggests that anxiety arises not simply from exposure itself, but from how performers interpret and internalize evaluative cues. From this perspective, evaluation anxiety may simultaneously undermine emotional stability while motivating increased preparation and control, depending on whether performers experience evaluation as threatening or as a signal of professional accountability. Support for Hypothesis 3 further indicates that online evaluation anxiety mediates the relationship between digital performance exposure and music performance anxiety, highlighting its central role in shaping emotional outcomes under conditions of sustained visibility. However, the persistence of a direct association between digital performance exposure and music performance anxiety, consistent with Hypothesis 4, suggests that digital visibility also exerts psychological effects beyond evaluative fear alone. This direct pathway may capture broader structural pressures linked to continuous observation, identity investment in performance, and the need to maintain a coherent professional self-presentation in public digital spaces. Taken in this light, music performance anxiety in digital contexts cannot be reduced to reactions to negative comments or explicit feedback. Rather, it appears to be embedded in the structural condition of visibility itself, where performers remain psychologically “on display” even in the absence of immediate evaluation. Digital platforms thus reshape the emotional ecology of performance by transforming anxiety from a situational response into a more enduring psychological condition—one that can operate as a source of strain for some performers while functioning as a form of adaptive vigilance for others, depending on individual orientation, experience, and the social meaning attached to evaluation.

### 5.2 Moderating roles of perfectionism and self-compassion in digital evaluation contexts

It should be noted that the moderating roles of perfectionism and self-compassion observed in this study do not imply that these traits exert stable or unidirectional effects across all contexts. Rather, the preceding analyses indicate that individual differences in self-regulation help explain variability in performers’ emotional responses to digital evaluation, and that such effects are contingent on both situational demands and the stage of the anxiety pathway involved. Specifically, the moderation results show that perfectionism operates at the earlier stage of the model by strengthening the association between digital performance exposure and online evaluation anxiety, such that performers with higher perfectionistic tendencies experience a steeper increase in evaluative concern as exposure intensifies. Under conditions of sustained digital visibility, the high internal standards and heightened attention to mistakes characteristic of perfectionism appear to increase sensitivity to evaluative cues, resulting in markedly different levels of evaluation anxiety even at comparable levels of exposure. Importantly, this pattern does not suggest that perfectionism is inherently maladaptive. Some performers may be able to maintain effective emotional regulation despite high standards, particularly when sufficient coping resources or professional experience are available. The present findings instead indicate that the emotional costs of digital exposure become more pronounced when elevated internal standards coincide with persistent external evaluation, a pattern consistent with the observed interaction in which exposure-related evaluation anxiety increases more sharply among highly perfectionistic performers. In contrast, self-compassion moderates a later stage of the anxiety pathway by weakening the association between online evaluation anxiety and music performance anxiety. This suggests that self-compassion does not primarily influence sensitivity to digital exposure itself, but rather affects how evaluative concern is regulated once it has been activated. Performers higher in self-compassion appear less likely to allow existing evaluation anxiety to escalate into more generalized performance anxiety. At the same time, this buffering effect should not be interpreted as a universal protective mechanism. In highly competitive digital performance contexts or situations where evaluative consequences are particularly salient, self-compassion may be insufficient to fully offset emotional strain, and excessive self-forgiveness may, in some cases, reduce motivation for performance improvement. Taken together, these findings do not support an idealized model of self-regulation, but instead delineate the limited and pathway-specific roles that perfectionism and self-compassion play in the formation of digital performance anxiety within the present sample and measurement framework. From an empirical standpoint, the emotional consequences of digital visibility depend not only on the intensity of exposure, but also on how performers regulate evaluative pressure at different stages of the anxiety process, underscoring the importance of maintaining caution when attributing explanatory primacy to any single psychological trait.

### 5.3 Differential patterns of digital performance anxiety across gender and performer identity

The multi-group analyses indicate that emotional responses to digital performance contexts are not uniform across performers. Although the overall structural model remains stable across gender and performer identity, differences in the strength of key pathways suggest that digital visibility and evaluation are experienced differently depending on performers’ social and professional positioning. These findings imply that digital evaluation does not exert a fixed level of psychological influence but operates through mechanisms whose salience varies across groups. At the gender level, the stronger associations observed among female performers between digital performance exposure and online evaluation anxiety, as well as between evaluation anxiety and music performance anxiety, point to greater sensitivity to evaluative cues in digitally mediated environments. This pattern does not necessarily indicate greater vulnerability. Rather, within the context of sustained online visibility, female performers may be more likely to attend to and internalize evaluative information, leading evaluative concerns to play a more central role in shaping anxiety responses. Under conditions where performances are continuously visible and comparable, such evaluative processing may become emotionally demanding. In contrast, among male performers, the direct association between digital performance exposure and music performance anxiety remains evident even when evaluation anxiety is taken into account. This suggests that, for this group, anxiety related to digital visibility may not be fully explained by concern over external evaluation. Instead, digital exposure may be experienced more directly as performance-related pressure or responsibility, independent of ongoing evaluative monitoring. This distinction indicates that the psychological pathways linking visibility to anxiety may differ in emphasis rather than in overall structure across gender groups.

Differences across performer identity further highlight the role of contextual stakes in shaping emotional responses to digital exposure. For professional musicians, the stronger link between digital performance exposure and online evaluation anxiety suggests that visibility is more readily interpreted as carrying tangible consequences. Given that online performance is often connected to reputation, career continuity, and future opportunities, evaluative cues in digital spaces may acquire heightened psychological significance. By contrast, student performers are more likely to encounter digital exposure within learning-oriented and transitional contexts. Although evaluation remains relevant, its emotional impact may be more situational and less likely to consolidate into persistent anxiety. Overall, these group-level differences do not alter the core pathway linking digital performance exposure, evaluation anxiety, and music performance anxiety. Instead, they clarify how the relative importance of specific psychological processes varies across performers. From an empirical perspective, these findings suggest that digital performance anxiety should be understood in relation to performers’ gender and career stage, as these factors shape how visibility and evaluation are perceived, interpreted, and emotionally integrated.

### 5.4 Contributions and limitations

The present study contributes to research on performance anxiety by integrating perspectives on digital visibility with models of emotional regulation, thereby offering a systematic account of how digitally mediated performance contexts reshape emotional experience. By clearly distinguishing digital performance exposure from general social media use, the study identifies a performance-specific psychological pathway through which sustained visibility and reliance on evaluation contribute to music performance anxiety, both directly and indirectly via online evaluation anxiety. This finding extends traditional approaches to music performance anxiety that have primarily emphasized situational stage-related stress, demonstrating that anxiety in digital environments is maintained not by isolated evaluative moments but by conditions of continuous observation. From a conceptual standpoint, the results suggest that performance anxiety in the digital era should not be understood solely as a transient emotional reaction, but rather as a more enduring emotional state structured by persistent exposure and ongoing feedback monitoring. In addition, the moderation analyses reveal differentiated regulatory pathways under digital visibility, indicating that perfectionism and self-compassion operate at distinct stages of the anxiety process. The multi-group analyses further show that while the overall model is structurally stable across gender and performer identity, the strength of key psychological pathways varies systematically with individual traits and social positioning. Together, these findings underscore that emotional experience in digital performance contexts is not merely an individual psychological phenomenon, but one embedded within technologically mediated systems of visibility and evaluation.

These theoretical and empirical contributions should nonetheless be interpreted in light of several limitations. Because participation in the study was voluntary and conducted through an online survey, the possibility of selection bias cannot be fully excluded. Performers experiencing higher levels of anxiety or greater sensitivity to evaluation may have been less willing to participate due to avoidance or emotional burden, potentially leading to an underrepresentation of individuals with more severe anxiety. To mitigate this risk, data collection was carried out through multiple channels, including universities, music institutions, and online performance communities, rather than relying on a single recruitment source. In addition, anonymity and non-evaluative instructions were used to reduce psychological barriers and encourage more open responding, although these measures cannot entirely eliminate self-selection effects. Another limitation concerns the absence of detailed information regarding participants’formal training background. This was a deliberate design choice rather than an oversight, as the study aimed to focus on psychological processes underlying digital performance anxiety rather than on identifying which performers are most susceptible. By centering the analysis on digital performance exposure, evaluation anxiety, and self-regulatory mechanisms, the model was intended to capture core emotional pathways without introducing extensive background stratification. However, this focus necessarily limits the extent to which differences related to competence level or professional experience can be examined. Moreover, the range of background variables collected was relatively narrow, and factors such as performance genre, frequency of online performance, or prior evaluative experiences were not included, which constrains the generalizability of the findings to other performance contexts. These limitations do not detract from the internal coherence or theoretical contribution of the study, but they do warrant caution when extending the results beyond samples and settings similar to those examined here. Rather than offering a definitive account of digital performance anxiety, the present study provides a conceptual and empirical framework that can guide future research, particularly studies incorporating developmental perspectives, ability-related factors, or longitudinal designs. From a practical standpoint, the findings also suggest that efforts to address performance anxiety in digital environments should move beyond strategies focused solely on reducing exposure or avoiding evaluation, and instead prioritize the cultivation of adaptive emotional regulation resources, especially self-compassion, to help performers maintain emotional stability under conditions of sustained visibility.

## 6 Conclusion

This study examined how digital performance exposure, online evaluation anxiety, and music performance anxiety (MPA) interact within contemporary digital performance environments, clarifying the emotional mechanisms through which visibility and evaluation shape performers’ experiences. Using structural equation modeling, the findings demonstrated that online evaluation anxiety mediates the relationship between digital performance exposure and MPA, indicating that digital feedback systems play a central role in transforming visibility into sustained emotional strain. Rather than functioning as a temporary reaction to isolated performance events, anxiety in digital contexts emerges as a more stable emotional condition maintained by continuous exposure and evaluative monitoring. The study further showed that individual differences in emotional regulation critically condition this process. Perfectionism amplified the impact of digital exposure on evaluation anxiety, whereas self-compassion buffered the translation of evaluative anxiety into performance-related anxiety, revealing two contrasting regulatory pathways under conditions of persistent visibility. Multi-group analyses additionally indicated that gender and professional identity shape the strength of these pathways, suggesting that emotional responses to digital evaluation are embedded in broader role expectations and occupational demands. Together, these findings extend existing models of music performance anxiety by situating them within digitally mediated environments and by demonstrating how technological visibility interacts with psychological susceptibility to structure emotional experience. Beyond theoretical contributions, the study underscores the practical importance of fostering adaptive emotional resources in music education and digital performance training. Supporting performers in developing self-awareness, reflective coping, and self-compassion may help mitigate the emotional costs of public exposure and sustain well-being in performance cultures increasingly shaped by visibility, comparison, and continuous evaluation.

## Data Availability

The raw data supporting the conclusions of this article will be made available by the authors, without undue reservation.

## Footnotes

1
https://www.wjx.cn
